# Circadian Variation in the Median Effective Dose of Epidural Ropivacaine for Labor Analgesia

**DOI:** 10.3389/fmed.2021.669264

**Published:** 2021-11-18

**Authors:** Jiali Deng, Changna Wei, Lin Liu, Jing Qian, Fei Xiao, Xinzhong Chen

**Affiliations:** ^1^Department of Anesthesia, Women's Hospital, Zhejiang University School of Medicine, Hangzhou, China; ^2^Department of Anesthesia, Jiaxing University Affiliated Women and Children Hospital, Jiaxing, China

**Keywords:** chronobiology, circadian rhythm, dose-response, epidural analgesia, labor analgesia, ropivacaine

## Abstract

**Background:** Labor pain perception has been demonstrated to exhibit a circadian rhythm with lower pain scores during the day compared with the night. This study aimed to determine and compare the median effective dose (ED_50_) of ropivacaine in parturients having epidural labor analgesia during the day vs. during the night.

**Methods:** The study group consisted of 60 nulliparous healthy parturients who were assigned to one of two groups according to the time they requested labor analgesia: Day Group (7:01 am to 7:00 pm) and Night Group (7:01 pm to 7:00 am). A bolus of.15% ropivacaine was administered epidurally and effective analgesia was defined as the attainment of a visual analog scale (VAS) pain score ≤ 10 mm within 30 min. The dose of ropivacaine for the first parturient in each group was 18 mg. The dose for each subsequent parturient was varied with increments or decrements of 3 mg based on the response of the previous subject. The ED_50_ was calculated using up-down sequential analysis. Probit regression was used to estimate the relative mean potency of ropivacaine between groups.

**Results:** The ED_50_ (mean [95% CI]) of ropivacaine was lower in the Day Group (17.9 [16.5–19.4] mg) than in the Night Group (20.9 [19.2–22.7] mg) (*P* = 0.003). The estimate of relative potency for ropivacaine for the Night Group vs. the Day Group was 0.85 (95% CI:0.56–0.98).

**Conclusions:** Under the conditions of this study, the dose requirement for epidural ropivacaine for labor analgesia was ~ 15% greater during the night than during the day.

**Clinical Trials Registration:** Chinese Clinical Trial Registry (No.: ChiCTR1900025269. http://www.chictr.org.cn/showprojen.aspx?proj=36993).

## Implication Statement

The dose requirement for epidural ropivacaine (AstraZeneca AB, Sweden) for labor analgesia in healthy parturients was ~15% greater during the night (7:01 pm to 7:00 am) than during the day (7:01 am to 7:00 pm).

## Introduction

Chronobiology, the circadian variation in biological rhythms, can influence many biological functions (1–4). Labor pain perception has been demonstrated previously to exhibit a circadian rhythm with lower pain scores during the day compared with the night (5, 6). Furthermore, the duration of epidural analgesia with ropivacaine during labor was found to be longer when administered during the day than the night (7). Accordingly, we considered that circadian rhythm in labor pain perception could influence epidural local anesthetic requirement. Therefore, we hypothesized that the dose requirement for ropivacaine would differ depending on whether epidural labor analgesia was administered during the day vs. the night. We designed the present study to determine and compare the median effective dose (ED_50_) of ropivacaine given for epidural labor in healthy parturients during the daytime vs. the night.

## Methods

After obtaining the approval from the Institutional Review Board of Jiaxing University Affiliated Women and Children Hospital, Jiaxing City, China (No. 2019Jun31), we registered the study in the Chinese Clinical Trial Registry (registration no., ChiCTR1900025269; the name of the principal investigator: Jiali Deng; date of registration: 18 August 2019) prior to the enrollment of first participant. The study was conducted in the delivery room of Jiaxing University Affiliated Women and Children Hospital Jiaxing City, China from 22 August 2019 to 22 October 2019. All parturients enrolled gave written informed consent.

The study recruited 60 full terms (≥37 weeks of gestation) nulliparous parturients with American Society of Anesthesiologists (ASA) physical status II who requested epidural analgesia in early labor (cervical dilation <3 cm). Parturients were only included if they had an uncomplicated pregnancy, spontaneous onset of labor, and had normal biological rhythm for rest and activity (normally woke in the morning and slept at night). Exclusion criteria were as follows: bodyweight <50 or > 90 kg, height <150 or >170 cm, administration of opioid or other modality of analgesia before epidural administration, rupture of membranes, contraindication to epidural anesthesia, allergy to ropivacaine, and inability or refusal to give informed consent.

Recruited parturients were assigned into one of two groups according to the time of day during which they requested epidural analgesia: Day Group (*n* = 30) for times between 7:01 am to 7:00 pm, and Night Group (*n* = 30) for times between 7:01 pm and 7:00 am. Two investigators were assigned to each time period. One was responsible for patient enrolment and drug preparation and the other, who was blinded to the dose of ropivacaine, was responsible for performing epidural analgesia and collecting data. All patients who met the inclusion criteria in each time period were eligible for enrolment in the study. However, for logistical considerations, no patient was enrolled if another patient had already been enrolled and study data collection had not been completed. Enrollment in each time period was ceased when the sample size for that group was achieved.

All parturients received an intravenous infusion of 250 ml warmed Ringer's lactate solution ~15 min before the epidural placement. Standard monitoring included non-invasive blood pressure, electrocardiography, pulse oximetry, and external cardiotocography. With the parturient in the left lateral position, an 18-gauge Tuohy needle was inserted into the epidural space at the estimated L_2−3_ vertebral interspace using a loss-of-resistance technique after skin infiltration with lidocaine. A multi-orifice epidural catheter was advanced cephalad 3 cm into the epidural space and secured. The parturient was then turned to the supine position with lateral uterine displacement. After careful aspiration to rule out subarachnoid or I.V. placement of the epidural catheter, a test dose of 3 ml of ropivacaine 0.15% (wt/vol) (4.5 mg) was injected through the catheter. Once a negative response was confirmed at 3 min after the injection of the test dose, a bolus of ropivacaine 0.15% (wt/vol) was then administered *via* the catheter as a bolus at a rate of ~1 ml/s. The dose for the first parturient in each group was set as 18 mg (12 ml) (including the test dose). The dose (the concentration of ropivacaine was fixed at 0.15% and the volume was the only variable factor) for each subsequent parturient was determined according to the response of the previous subject using an up-down sequential allocation technique as described by Dixon and Massey (8). Pain scores were assessed using a 100-mm visual analog scale (VAS) (0 = no pain, 100 = worst pain imaginable) at the peak of a uterine contraction. Effective analgesia was defined as the attainment of a VAS pain score ≤ 10 mm within 30 min after completion of epidural injection. Effective analgesia directed a decrement of 3 mg of ropivacaine for the next parturient assigned to that group whereas ineffective analgesia directed an increment of 3 mg. Parturients who had ineffective analgesia received 10 ml of ropivacaine 0.20% (wt/vol) as a rescue. If VAS still remained > 10 mm after the injection of the rescue dose, the parturient was excluded from the study. Parturients were also excluded from the study if, within 30 min of study drug administration, their cervix became fully dilated or they required Cesarean delivery. When such patients were excluded, the next parturient received the same volume of ropivacaine as the previous subject. At 30 min after injection of the initial dose of ropivacaine, parturients received an infusion of a mixture of ropivacaine 0.1% (wt/vol) and 0.5 μg·ml^−1^ sufentanil (Humanwell Pharmaceutical Co., Ltd, china) *via* patient-controlled epidural analgesia according to standard institutional practice (bolus, 8 ml; lockout interval, 15 min; background infusion rate, 8 ml·h^−1^).

We collected the following data before epidural placement, at 5-min intervals for 30 min. Then, at 30-min intervals until delivery of the baby we collected the following: VAS pain score; upper dermatomal level of sensory block assessed by loss of cold discrimination using alcohol-soaked cotton; motor block according to a modified Bromage scale (0 = no motor loss, 1 = inability to flex hip, 2 = inability to flex hip and knee, 3 = inability to flex hip, knee, and ankle); blood pressure and heart rate. Onset time of labor analgesia was defined as the time between the completion of the initial bolus of epidural ropivacaine until a VAS pain score ≤ 10 mm was attained. The patient-controlled epidural analgesia (PCEA) of patient requirements and manual bolus were recorded. We also recorded maternal demographic data (age, weight, height, gestation, educational level), side effects (hypotension, shivering, pruritus, bradycardia, respiratory depression, nausea, and vomiting), labor duration, and mode of delivery, fetal bradycardia, and neonatal Apgar scores. Hypotension was defined as a decrease in systolic BP to <20% of the baseline value and was treated with intravenous fluid or phenylephrine 40 μg. Bradycardia was defined as a heart rate <50 beats/min and was treated with intravenous atropine 0.5 mg. Fetal bradycardia (defined as a heart rate <110 bpm). Respiratory was defined as a respiratory rate <8 breaths/min or SpO_2_ <90%.

### Statistical Analysis

The sample size of 30 was determined according to the results of previous studies that recommended that 20–40 subjects were sufficient to provide a stable estimate of the ED_50_ calculated by the Dixon up–and–down method for most realistic scenarios (9, 10). Statistical analyses were performed using IBM SPSS for Windows version 22 (IBM Corp, Armonk, NY, USA) and GraphPad Prism version 5 (GraphPad Software Inc, San Diego, CA, USA). *P* < 0.05 was considered to be statistically significant.

For continuous variables, the D'Agostino & Pearson normality test was used to test the normality of distribution. Variables with normal distribution were presented as *M* ± *SD* and analyzed with Student's *t-test*. Variables with non-normal distribution were presented as the median and interquartile range (IQR) and were analyzed with the Mann-Whitney U test. Categorical variables were presented as numbers (%) and were analyzed using the chi-square test. Values for the ED_50_ of epidural ropivacaine were determined by calculating the mean of the midpoints of pairs of ropivacaine doses administered in successive parturients in which a case of ineffective analgesia was followed by a case of effective analgesia, or a case of effective analgesia was followed by a case of ineffective analgesia (turning points) according to the modified up-and-down allocation method as described previously (11–13). The 95% CI and *SD* for the ED_50_ values were calculated using the method suggested by Choi (13). In addition, we used Probit regression analysis as a backup and sensitivity test by analyzing tallied numbers of effective and ineffective analgesia for each dose category for each group (9). In the latter analysis, estimates of the ED_50_ of the ropivacaine in each group were obtained and the difference between the two groups was quantified by calculating the relative mean potency with 95% CI as previously described (14).

## Results

**Figure 1:**
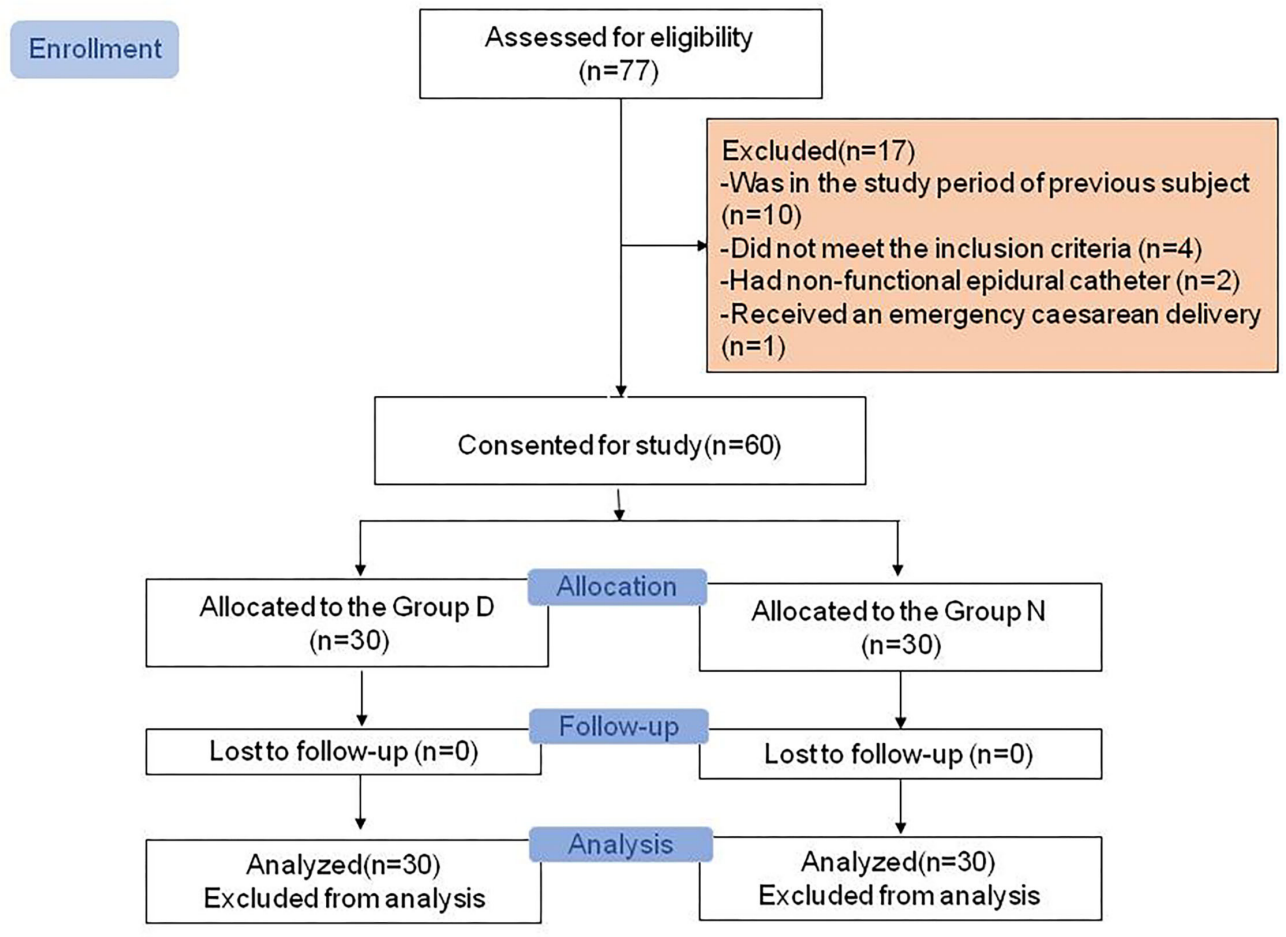
Consolidated Standards of Reporting Trials (CONSORT) diagram showing patient recruitment and study flow.

The Consolidated Standards of Reporting Trials (CONSORT) diagram of the study is presented in Figure 1. A total of 77 parturients was assessed for eligibility, of whom 10 were not enrolled because another patient had already been enrolled and study data collection had not been completed, four did not meet the inclusion criteria, two had a nonfunctional epidural catheter, and one required emergency Cesarean delivery during the study period. The remaining 60 parturients (30 in the Day Group and 30 in the Night Group) completed the study and had data analyzed. Demographic and obstetric characteristics did not differ between the two groups (all *P* > 0.05; Table 1).

**Table 1:** Demographic and obstetric characteristics.

	**Day Group**	**Night Group**	***P***
	**(*n* = 30)**	**(*n* = 30)**	
Age (years)	27.5 ± 3.5	26.5 ± 2.8	0.26
Height (cm)	159.9 ± 4.0	160.8 ± 4.3	0.42
Weight (kg)	63.9 ± 7.7	66.7 ± 8.3	0.19
Gestational age (wk)	39.7 ± 1.0	39.5 ± 0.9	0.50
**Educational level**			
Junior college or lower (n)	15 (50%)	15 (50%)	> 0.99
Undergraduate (n)	15 (50%)	14 (47%)	0.80
Graduate or higher (n)	0	1 (3%)	> 0.99

*Data are expressed as M ± SD, median (interquartile range), or number (%)*.

**Figure 2:**
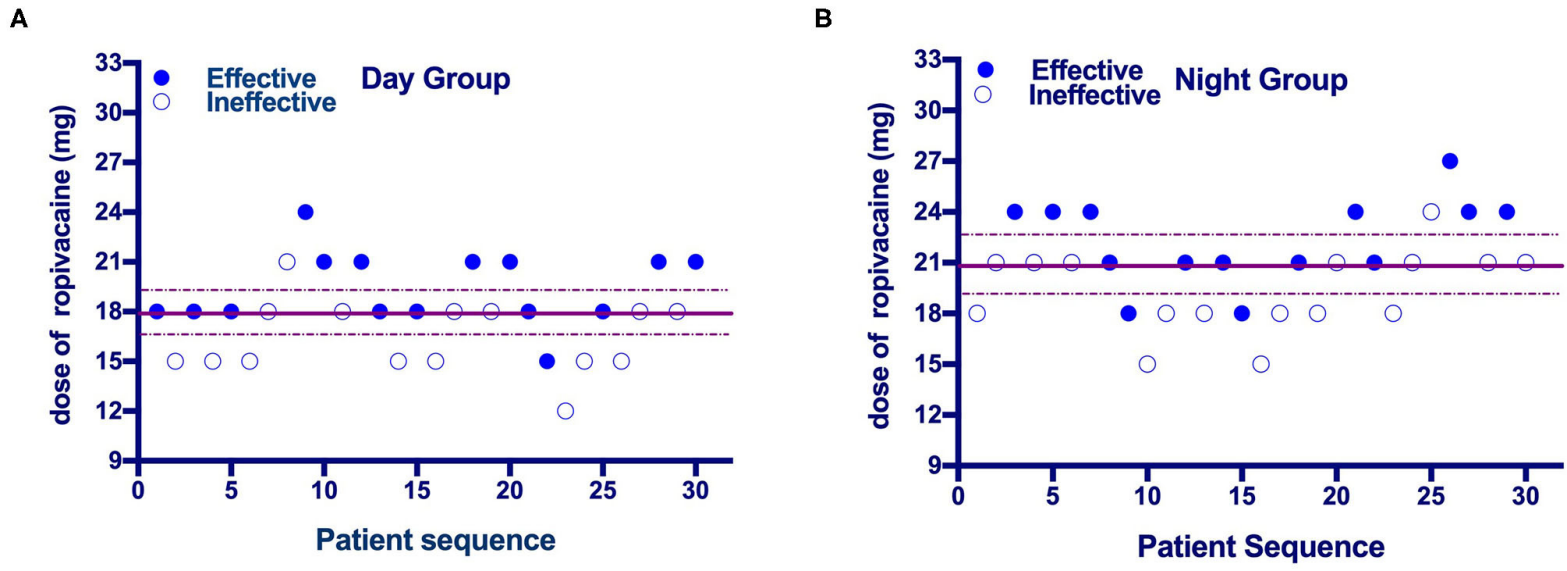
**(A,B)** Individual response to epidural ropivacaine at a corresponding dose (mg). The median effective dose (ED_50_) of epidural ropivacaine for labor analgesia was 17.9 (95% CI: 16.5 to 19.4) mg in the Day Group vs. 20.9 (95% CI: 19.2 to 22.7) mg in the Night Group. Solid lines represent the ED_50_ values and dashed lines represent the 95% CI.

**Figure 3:**
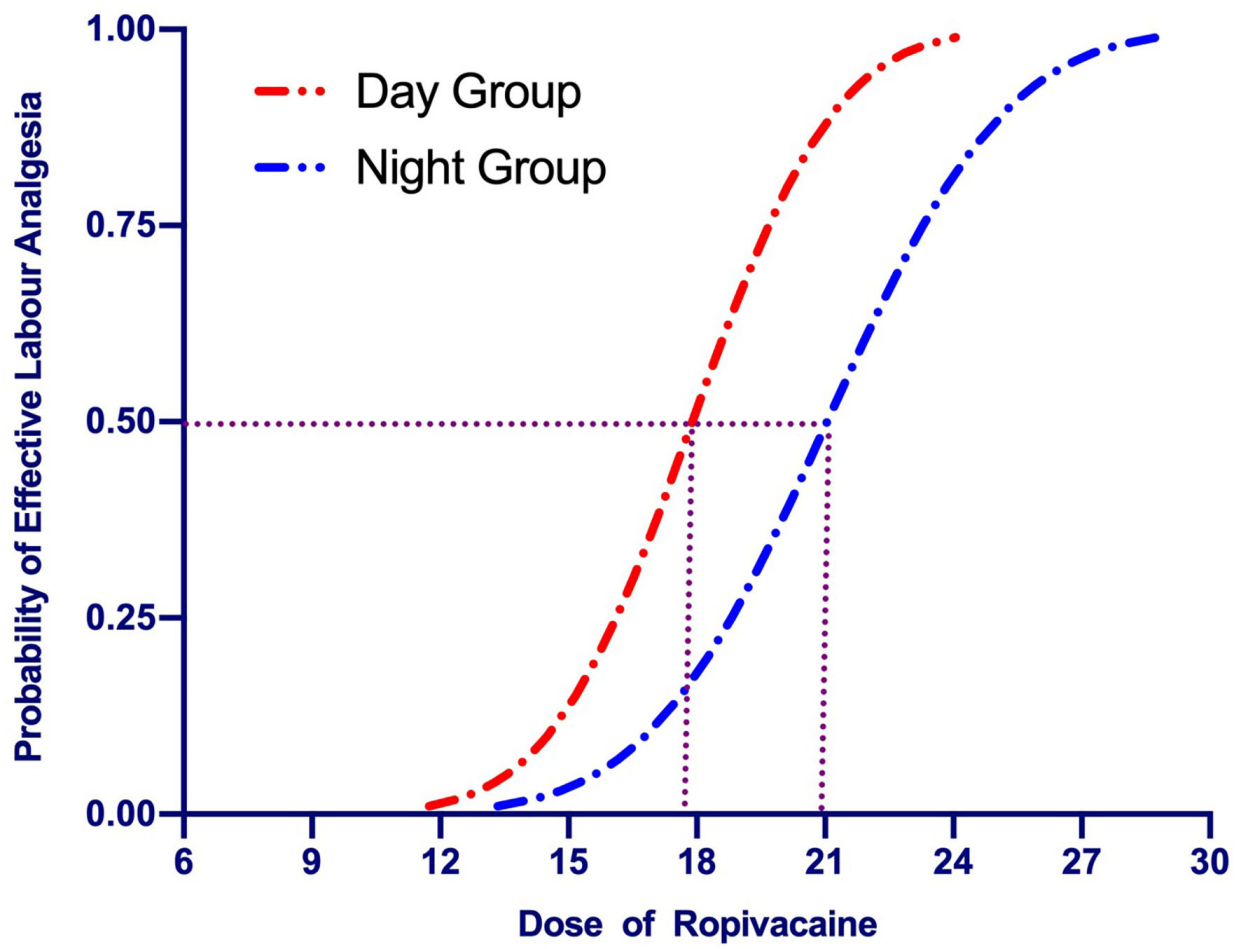
Dose-response curves of epidural ropivacaine for labor analgesia plotted from estimated probabilities of effective response (1% to 100) vs. the corresponding dose of the initial bolus derived from probit regression analysis. The median effective dose (ED_50_) of epidural ropivacaine for labor analgesia was 17.9 (95% CI: 15.8–20.0) mg in the Day Group vs. 21.0 (95% CI: 18.9–23.4) mg in the Night Group.

The up-down sequences are shown in Figures 2A,B. The calculated value for ED_50_ of ropivacaine was lower in the Day Group (17.9 mg [95% CI, 16.5–19.4]) compared with the Night Group (20.9 mg [95% CI, 19.2–22.7]) (*P* = 0.003). The ED_50_ values calculated using probit regression were 17.9 mg (95% CI, 15.8–20) in the Day Group and 21 mg (95% CI, 18.9–23.4) in the Night Group. The estimate of relative mean potency for ropivacaine in the Night Group vs. the Day Group was.85 (95% CI,0.56–0.98). The ED_90_ values calculated using probit regression were 21.3 (95% CI, 19.5–28.8) mg in the Day Group and 25.3 mg (95% CI, 2–37.6) in the Night Group. Dose-response curves derived from probit regression analysis are shown in Figure 3.

Cervical dilation at the time when parturients were recruited was not different between groups (Table 2). However, the mean VAS pain score at this time was lower in the Day Group compared with the Night Group (71.8 ± 3.6 vs. 82.5 ± 3.2, *P* < 0.001) (Table 2). There were no differences between the groups in the onset time of analgesia, the maximum sensory block level, and the maximum modified Bromage score during the first 30 min after epidural administration (all *P* > 0.05) (Table 2). There were no significant differences in the PCEA requirements of the patients and manual bolus (all *P* > 0.05) during labor. There were no differences between the groups in the incidences of nausea, vomiting, shivering, pruritus, hypotension, bradycardia, or respiratory depression (Table 2). There were no differences between the groups in the newborn umbilical artery pH, Apgar score at 1 and 5 min, and the incidences of fetal bradycardia (Table 3).

**Table 2:** Maternal outcomes (including side effects).

	**Day Group**	**Night Group**	***P***
	**(*n* = 30)**	**(*n* = 30)**	
Baseline visual analog scale pain score (mm)	71.8 ± 3.6	82.5 ± 3.2	<0.001
Onset of analgesia (min)	17.2 ± 3.7	17.9 ± 3.6	0.60
Maximum sensory block	T10	T10	0.64
level	[T8–T12]	[T8–T11]	
**Maximum modified Bromage score**			
0-1-2-3 (n)	30-0-0-0	30-0-0-0	0.99
PCEA requests (n)	3 (3, 4)	4 (3, 4)	0.07
No. of manual bolus [n (%)]	1 (3.3)	2 (6.6)	0.554
Cervical dilation at inclusion (cm)	2 (2~2)	2 (2~2)	> 0.99
Duration of first stage of labor (min)	525.7 ± 165.1	597.0 ± 162.0	0.11
Duration of second stage of labor (min)	76.0 ± 165.1	66.8 ± 31.9	0.40
Cesarean delivery	2 (6.7%)	2 (6.75)	> 0.99
Nausea or vomiting (n)	2 (6.7%)	1 (3.3%)	0.55
Shivering (n)	1 (3.3%)	1 (3.3%)	0.99
Pruritus (n)	0	0	0.99
Hypotension (n)	0	0	0.99
Bradycardia (n)	0	0	0.99
Respiratory depression (n)	0	0	0.99

*Data are presented as mean ± standard deviation or median [interquartile range] or number (%)*.

**Table 3:** Neonatal outcomes.

	**Day Group**	**Night Group**	***P***
	**(*n* = 30)**	**(*n* = 30)**	
Fetal bradycardia (*n*)	1 (3.3)	0 (0)	0.313
Apgar score at 1 min	10.0 [9.8–10.0]	10.0 [10.0–10.0]	0.42
Apgar score at 5 min	10.0 [10.0–10.0]	10.0 [10.0–10.0]	0.98
Umbilical arterial pH	7.3 [7.3–7.3]	7.3 [7.2–7.3]	0.53

*Data are presented as mean ± standard deviation or median [interquartile range] or number (%)*.

## Discussion

In this prospective, up-down sequential allocation study, we found diurnal variation in the median ED_50_ of epidural ropivacaine for labor analgesia in nulliparous parturients. Our findings suggest that the dose requirement for epidural ropivacaine for effective labor analgesia is ~15% higher when it is administered during the night compared with during the day.

Several previous studies have investigated the dose requirement for epidural ropivacaine for labor analgesia under various circumstances but few have considered a time of day as a factor (15–19). The study of Debon et al. (7) investigated the chronobiology of epidural ropivacaine in laboring parturients and found that its duration of action was greater by up to 28% in the diurnal period compared with the nocturnal period. However, these authors did not investigate whether the dose required to achieve analgesia differed between periods. To the best of our knowledge, our present study is the first to quantify the effect of the time of day on the dose requirement of epidural ropivacaine for labor analgesia.

Several factors may have contributed to our findings. One of the most important may be inherent biological circadian rhythms which may influence physiological and/or pharmacological processes. Circadian variations have been reported for almost all major physiological functions, the pharmacokinetics and pharmacodynamics of several medications, and time of day are considered important factors influencing anesthesia and pain treatment (1–4, 6). In our study, we found that the mean VAS pain scores at the time when parturients requested epidural analgesia were higher in the Night Group than in the Day Group. This was despite no difference between groups in obstetric and other factors such as cervical dilation, parity, spontaneous or pharmacologically induced labor, rupture of membranes, duration of labor, and educational level. This finding is consistent with the results of previous studies that also found that labor pain scores were higher at night than during the day (5, 6, 20). Together, these studies are highly indicative of the existence of circadian variation in labor pain. Greater baseline pain at the time of request for epidural analgesia could account for the greater dose requirement for ropivacaine at night.

There are several possible mechanisms that may underline the circadian variation described above. First, sleep deprivation at night could reduce pain thresholds and enhance pain sensitivity (21). Moreover, sleep deprivation can interfere with analgesic treatments involving opioid and serotoninergic mechanisms of action (22). Second, the release of antinociceptive hormones and peptides such as adrenocorticotropic hormone (ACTH), cortisol, β-endorphin, and NR2B may be important. These are increased during pregnancy resulting in elevation of pain tolerance and have been found to exhibit circadian rhythms with higher plasma concentrations in the daytime (23–26). Third, there may be circadian influences on the action and kinetics of epidural ropivacaine. For example, circadian variation in membrane permeability to local anesthetics was described in a study of erythrocyte penetration by bupivacaine, etidocaine, and mepivacaine in mice (27).

In addition to biological factors, it is possible that external and environmental factors not identified by our study may have contributed to the circadian variation observed. For example, this might include influences related to shifts of nursing, anesthetic, and other healthcare staff. During the day, patients may be distracted by external factors such as interactions with visitors and electronic communication. Such factors may exhibit or influence diurnal rhythms but may not have a strictly biological basis (28).

Regardless of the underlying mechanism, our results have relevance for clinical practice in obstetric analgesia. To minimize potential side effects of epidural local anesthetics in labor it is desirable to use the minimum dose required to provide adequate analgesia. Our main finding of a 15% difference in epidural ropivacaine requirement between day and night may inform the choice of initial dose according to the time of day.

Our study has several limitations. First, we investigated the dose requirement of epidural ropivacaine for labor analgesia in only two time periods in the day, the cutoffs for which were arbitrarily chosen. Second, we were not able to include all eligible subjects per period because of logistical limitations which potentially could have created unidentified selection bias. Third, because of the strict inclusion and exclusion criteria, our results may not be generalizable to all cases, for example, the mean BMI in this study was only 25 and 26 in the two groups. Finally, this study was conducted in the context of a university hospital in China, and we only investigated healthy nulliparous women in early spontaneous labor without augmentation. The non-biological factors related to this specific environment may influence the external validity of our findings. We recommend that further similar studies be conducted in other units and in other countries to confirm our findings in other contexts such as in multiparous women with or without augmentation, and for maintenance of analgesia in addition to initiation of analgesia using programmed intermittent epidural bolus (PIEB) regimens.

In summary, under the conditions of this study, the dose requirement for epidural ropivacaine for labor analgesia was ~15% greater during the night than during the day.

## Data Availability Statement

The raw data supporting the conclusions of this article will be made available by the authors, without undue reservation.

## Ethics Statement

The studies involving human participants were reviewed and approved by Institutional Review Board of Jiaxing University Affiliated Women and Children Hospital, Jiaxing City, China (No. 2019Jun31). The patients/participants provided their written informed consent to participate in this study.

## Author Contributions

JD helped in designing and conducting the study and writing the manuscript. CW helped in conducting the study and writing the manuscript. LL and JQ helped in conducting the study. FX helped in designing the study and data analysis. XC helped in designing the study and writing the manuscript. All authors contributed to the article and approved the submitted version.

## Funding

This work was supported by grants from the National Natural Science Foundation of China (NSFC) (Nos: 81471126 and 81870868).

## Conflict of Interest

The authors declare that the research was conducted in the absence of any commercial or financial relationships that could be construed as a potential conflict of interest.

## Publisher's Note

All claims expressed in this article are solely those of the authors and do not necessarily represent those of their affiliated organizations, or those of the publisher, the editors and the reviewers. Any product that may be evaluated in this article, or claim that may be made by its manufacturer, is not guaranteed or endorsed by the publisher.

## References

[1] Pears S Makris A Hennessy A. The chronobiology of blood pressure in pregnancy. Pregnancy Hypertens. (2018) 12:104–9. 10.1016/j.preghy.2018.04.002 29674188

[2] Abele SH MK Medeiros D Silver AC. Time is on the Immune System's Side, Yes it is [review]. Yale J Biol Med. (2019) 92:7. 31249483 PMC6585517

[3] Haus E Cusulos M Sackett-Lundeen L Swoyer J. Circadian variations in blood coagulation parameters, alpha-antitrypsin antigen and platelet aggregation and retention in clinically healthy subjects. Chronobiol Int. (1990) 7:203–16. 10.3109/07420529009056976 2125246

[4] Montaigne D Marechal X Modine T Coisne A Mouton S Fayad G. Daytime variation of perioperative myocardial injury in cardiac surgery and its prevention by Rev-Erbα antagonism: a single-centre propensity-matched cohort study and a randomised study. Lancet. (2018) 391:59–69. 10.1016/S0140-6736(17)32132-3 29107324

[5] Desai S Leong SB Yvonne L Sia A. Chronobiology of parturients receiving neuraxial labour analgesia with ropivacaine and fentanyl: a prospective cohort study. Int J Obstet Anesth. (2009) 18:43–7. 10.1016/j.ijoa.2008.07.012 19046873

[6] Aya AG Vialles N Mangin R Robert C Ferrer JM Ripart J. Chronobiology of labour pain perception: an observational study. Br J Anaesth. (2004) 93:451–3. 10.1093/bja/aeh223 15247110

[7] Debon R Chassard D Duflo F Boselli E Bryssine B Allaouchiche B. Chronobiology of epidural ropivacaine: variations in the duration of action related to the hour of administration. Anesthesiology. (2002) 96:542–5. 10.1097/00000542-200203000-00006 11873025

[8] Dixon WJ. Staircase bioassay: the up-and-down method. Neurosci Biobehav Rev. (1991) 15:47–50. 10.1016/S0149-7634(05)80090-9 2052197

[9] Pace NL Stylianou MP. Advances in and limitations of up-and-down methodology: a précis of clinical use, study design, and dose estimation in anesthesia research. Anesthesiology. (2007) 107:144–52. 10.1097/01.anes.0000267514.42592.2a 17585226

[10] Pan PH Lee S Harris L. Chronobiology of subarachnoid fentanyl for labor analgesia. Anesthesiology. (2005) 103:595–9. 10.1097/00000542-200509000-00023 16129985

[11] Choi SC. An investigation of Wetherill's method of estimation for the up-and-down experiment. Biometrics. (1971) 27:961–70. 10.2307/2528831 5138939

[12] Fu F Chen X Feng Y Shen Y Feng Z Bein B. Propofol EC50 for inducing loss of consciousness is lower in the luteal phase of the menstrual cycle. Br J Anaesth. (2014) 112:506–13. 10.1093/bja/aet383 24285693

[13] Choi SC. Interval estimation of the LD50 based on an up-and-down experiment. Biometrics. (1990) 46:485–92. 10.2307/2531453 2364133

[14] Xiao F Wei C Chang X Zhang Y Xue L Shen H. A prospective, randomized, double-blinded study of the effect of intravenous ondansetron on the effective dose in 50% of subjects of prophylactic phenylephrine infusions for preventing spinal anesthesia-induced hypotension during cesarean delivery. Anesth Analg. (2020) 131:564–9. 10.1213/ANE.0000000000004534 31725021

[15] Meister GC D'Angelo R Owen M Nelson KE. Gaver R. A comparison of epidural analgesia with 0125% ropivacaine with fentanyl vs. 0125% bupivacaine with fentanyl during labor. Anesth Analg. (2000) 90:632–7. 10.1097/00000539-200003000-00024 10702449

[16] Aveline C El Metaoua S Masmoudi A Boelle PY Bonnet F. The effect of clonidine on the minimum local analgesic concentration of epidural ropivacaine during labor. Anesth Analg. (2002) 95:735–40. 10.1213/00000539-200209000-00037 12198062

[17] Boulier V Gomis P Lautner C Visseaux H Palot M Malinovsky JM. Minimum local analgesic concentrations of ropivacaine and levobupivacaine with sufentanil for epidural analgesia in labour. Int J Obstet Anesth. (2009) 18:226–30. 10.1016/j.ijoa.2009.02.002 19464878

[18] Qeng Q Yang Z Zhang W Wu X. Comparison of median effective concentration of ropivacaine in multiparas or primiparas during epidural labor analgesia: STROBE compliant. Medicine (Baltimore). (2020) 99:e18673. 10.1097/MD.0000000000018673 31895835 PMC6946190

[19] Palm S Gertzen W Ledowski T Gleim M Wulf H. Minimum local analgesic dose of plain ropivacaine vs. ropivacaine combined with sufentanil during epidural analgesia for labour. Anaesthesia. (2001) 56:526–9. 10.1046/j.1365-2044.2001.02050.x 11412157

[20] Vieira WS Hidalgo MP Torres Ida S Caumo W. Biological rhythms of spinal-epidural labor analgesia. Chronobiol Int. (2010) 27:865–78. 10.3109/07420521003721914 20560716

[21] Larson RA Carter JR. Total sleep deprivation and pain perception during cold noxious stimuli in humans. Scand J Pain. (2016) 13:12–6. 10.1016/j.sjpain.2016.05.037 27867438 PMC5111796

[22] Lautenbacher S Kundermann B Krieg JC. Sleep deprivation and pain perception. Sleep Med Rev. (2006) 10:357–69. 10.1016/j.smrv.2005.08.001 16386930

[23] Lindow SW Newham A Hendricks MS Thompson JW van der Spuy ZM. The 24-hour rhythm of oxytocin and beta-endorphin secretion in human pregnancy. Clin Endocrinol (Oxf). (1996) 45:443–6. 10.1046/j.1365-2265.1996.8290840.x 8959083

[24] Xia T Cui Y Qian Y Chu S Song J Gu X. Regulation of the NR2B-CREB-CRTC1 signaling pathway contributes to circadian pain in murine model of chronic constriction injury. Anesth Analg. (2016) 122:542–52. 10.1213/ANE.0000000000000991 26440419

[25] Hamra JG Kamerling SG Wolfsheimer KJ Bagwell CA. Diurnal variation in plasma ir-beta-endorphin levels and experimental pain thresholds in the horse. Life Sci. (1993) 53:121–9. 10.1016/0024-3205(93)90659-Q 8515686

[26] Whipple B Josimovich JB Komisaruk BR. Sensory thresholds during the antepartum, intrapartum and postpartum periods. Int J Nurs Stud. (1990) 27:213–21. 10.1016/0020-7489(90)90036-I 2379982

[27] Bruguerolle B Prat M. Temporal variations in the erythrocyte permeability to bupivacaine, etidocaine and mepivacaine in mice. Life Sci. (1989) 45:2587–90. 10.1016/0024-3205(89)90243-9 2615557

[28] Shafer SL Lemmer B Boselli E Boiste F Bouvet L Allaouchiche B. Pitfalls in chronobiology: a suggested analysis using intrathecal bupivacaine analgesia as an example. Anesth Analg. (2010) 111:980–5. 10.1213/ANE.0b013e3181dd22d4 20442259

